# Normal sequential changes in neutrophil CD64 expression after total joint arthroplasty

**DOI:** 10.1007/s00776-013-0451-9

**Published:** 2013-08-14

**Authors:** Narutaka Katoh, Jinju Nishino, Keita Nishimura, Chisato Kawabata, Yuuko Hotta, Toshihiro Matsui, Shigeru Nakamura, Takashi Matsushita

**Affiliations:** 1 Department of Orthopaedic Surgery, Teikyo University School of Medicine, 2-11-1 Kaga, Itabashi-ku, Tokyo, Japan; 2 Nishino Clinic, Orthopedics and Rheumatology, Tokyo, Japan; 3 Department of Rheumatology, Sagamihara National Hospital, National Hospital Organization, Kanagawa, Japan

## Abstract

**Background:**

Neutrophil CD64 has been reported to be a sensitive and specific infection marker. Its measurement is thus considered to be useful in early diagnosis of post-operative periprosthetic infection. However, even its normal sequential changes after non-infectious total joint arthroplasty have remained ambiguous. Accordingly, we analyzed 2-week sequential neutrophil CD64 expression changes after total joint arthroplasty in order to clarify its normal postoperative kinetics.

**Patients and method:**

From 41 patients who underwent primary total joint arthroplasties, peripheral blood samples were obtained at 1 day before (baseline) and 1, 3, 5, 7, and 14 days after surgery, and CD64 expression per cell was quantitatively measured. C-reactive protein (CRP) level, erythrocyte sedimentation rate (ESR) and white blood cell count (WBC) were simultaneously measured.

**Results:**

No cases of postoperative local infection were observed. Levels of CD64 significantly elevated from day 1, peaked at day 3, and decreased significantly following day 5. Statistical analysis confirmed that significant differences existed between the baseline level and the levels at days 1 and 3, while no significant differences existed between the baseline level and those at days 5, 7 or 14. In 17 patients, CD64 peaked at over 2,000 molecules/cell, the level reported to be a cutoff value for distinguishing infection. Multiple regression analysis showed that the sole parameter of baseline CD64 level significantly explained the peak CD64 level. Postoperative CD64 peaks ranged from 1.6 to 2.7 times (median 1.9) the baseline levels. CRP, ESR and WBC also showed rapid elevations and all but WBC remained significantly higher than baseline at day 14.

**Conclusion:**

CD64 levels rise significantly, peaking within about 3 days following normal total joint arthroplasty, but decrease rapidly to near baseline within about 5 days. The data obtained can be expected to form a possible basis for early diagnosis of postoperative periprosthetic infection.

## Introduction

Periprosthetic joint infection following total joint arthroplasty is still one of the most devastating complications. Once an infection occurs, it inflicts a significant financial burden as well as physical and psychological morbidity on patients. Early infection after surgery particularly requires fast and accurate diagnosis because a delay in treatment appears to be the most detrimental factor to a successful outcome. Nevertheless, its diagnosis often fails since there have been no adequate procedures for detecting such infection at an early stage after surgery. For example, findings of local clinical symptoms such as local heat or swelling, or elevations of levels in laboratory markers such as white blood cell count (WBC), C-reactive protein (CRP) level or erythrocyte sedimentation rate (ESR), may overlap with physiological responses to the surgical stress.

FcγRI (CD64), an Fc receptor for IgG, is known to play a role in antibody-dependent cytotoxicity, clearance of immune complexes and phagocytosis of targets opsonized with IgG [1, 2]. It mediates the release of proinflammatory cytokines such as interleukin (IL) -1 and -6, and tumor necrosis factor-α [3]. Constitutively, CD64 is expressed on macrophages and monocytes, and is upregulated on neutrophils as a physiological response to microbial wall components, complement split products, and some cytokines such as interferon-γ (IFN-γ), IL-8 and -12, and granulocyte colony stimulating factor (G-CSF) [4, 5].

Of note, CD64 expression on the surface of neutrophils can be induced by viruses and mycobacteria as well as bacteria [6, 7]. Consequently, several recent reports indicated that the neutrophil CD64 is a sensitive and specific marker of systemic infection and local infection [1, 2, 6, 8–11]: Matsui et al. [1] reported that a cutoff value for CD64 for distinguishing systemic infection was 2,000 molecules/cell, and Tanaka et al. [2] showed that CD64 levels in patients with musculoskeletal local infection were significantly higher than those in patients without infection. Based on these studies, the measurement of neutrophil CD64 expression was considered to be a strong candidate for use in the early diagnosis of post-operative periprosthetic infection.

However, previous studies focusing on neutrophil CD64 expression during the early postoperative period are very few in number. Fjaertoft et al. [12] investigated the changes of semi-quantitative expression levels for 3 days after total hip arthroplasty (THA) in 12 cases. They showed that the expressions increased for the observed 3 days. Strohmeyer et al. [13] indicated that the expression levels, obtained by fully quantitative measurement, peaked at day 2 after cardiopulmonary bypass surgery, and Kolackova et al. [14] reported that fully quantitative CD64 levels significantly elevated but returned to baseline by day 7 after cardiac surgeries.

Those previous studies indicated that CD64 level will elevate during the early phase after surgery. However, the first of the above authors only investigated a small number of subjects and for only 3 days using just semi-quantitative means, data obtained by which cannot be compared objectively among multi-centers. The other authors carried out their investigations in cardiac surgeries that were completely different from total joint arthroplasty. Thus, the normal sequential changes after non-infectious total joint arthroplasty have remained absolutely ambiguous. If they could be accurately grasped, the data obtained could form a basis for an accurate diagnosis of early periprosthetic infection after surgery.

In the current study, therefore, in order to clarify normal kinetics of neutrophil CD64 expression after total joint arthroplasty, fully quantitative measurement of the expression was made over a period of 2 weeks following the surgery in a larger number of subjects.

## Materials and methods

### Patients

Included in this study were patients who gave their written informed consent and who underwent total knee arthroplasties (TKA) or THA for treatment of their conditions between January and June in 2008 at a University Hospital. The study was approved by the local ethics committees.

Enrolled in this study were 54 patients who underwent TKA or THA. Among the 54 cases, 13 were excluded from further analysis, 11 because of comorbidities: chronic infectious disease (chronic hepatitis, chronic respiratory infection), malignant lymphoma and post-operative deep venous thrombosis, and 2 because they were revision cases.

Finally, 41 patients were assessed. Their diagnoses were 17 knee- and 18 hip- osteoarthritis, and 2 knee- and 4 hip-aseptic necrosis. Nineteen TKA (cemented type), 22 THA (uncemented type) were undergone. In all cases, prophylactic antibiotics were routinely administered during the surgeries and for 3 days following them. Deep drainages with suction tubes were also applied for 48 h after surgery.

Clinical and demographic characteristics of the patients at baseline are presented in Table 1. There were no significant differences between the 41 patients assessed and 13 patients excluded. Surgical details of the assessed TKA and THA are presented in Table 2.

**Table 1:** Clinical and demographic characteristics at baseline

	Enrolled patients	Assessed patients	Excluded patients	*p* value
*n* (patients)	54	41	13	
Gender				
Female/male	43/11	33/8	10/3	0.962
Age (years)				
Median [IQR]	66.8 [58.3–75.0]	67.8 [58.3–75.0]	67.6 [56.0–77.5]	0.816
Diagnosis				
Knee osteoarthritis	21	17	4	0.973
Hip osteoarthritis	25	18	7	
Knee- or hip- necrosis	8	6	2	
Surgery				
Total knee arthroplasty	23	19	4	0.613
Total hip arthroplasty	31	22	9

**Table 2:** Surgical details in TKA and THA

	TKA	THA
*n* (patients)	19	22
Operative duration (min)	120 [110–132]	69 [60–80]
Blood loss in surgery (ml)	40 [10–67]	281 [152–433]
Postoperative bleeding (from drain) (ml)	845 [600–1,021]	269 [149–387]

*TKA* total knee arthroplasty, *THA* total hip arthroplasty

### Measurement of CD64 expression

The peripheral blood samples used for CD64 measurement were obtained at 1 day before (baseline) and at days 1, 3, 5, 7, and 14 after the surgeries.

Levels of neutrophil CD64 expressions per cell were quantitatively measured, as was originally described by Matsui et al. [1]. Briefly, after staining all of the blood with QuantiBRITE CD64PE/CD45PerCP (Beckton-Dickinson, San Jose, CA, USA), the granulocyte population was gated by the CD45/side-scatter profile. The CD64 levels were analyzed using a FACScan flow cytometer (Beckton-Dickinson) calibrated with QuantiBRITE PE beads (Beckton-Dickinson).

CRP level, ESR and WBC were also simultaneously measured using the same blood samples.

### Statistical analysis

The measurement variables are presented as the median and interquartile range [IQR]. Comparison among the continuous variables was carried out using one-way ANOVA followed by Dunnett’s procedure for post-test comparison. Correlations among the variables were evaluated by calculated Spearman’s rank correlation coefficient. To select the variables that were relevant to CD64 level, a stepwise regression analysis was performed. Statistical significance was set at *p* < 0.05. All analyses were conducted using the SigmaStat statistical program (version 18, SPSS Science, Chicago, IL, USA).

## Results

### Clinical results

No cases complicated with post-operative local infection were observed for the full investigated 2 weeks following the surgeries, nor for the ensuing period to the end of June 2009.

### Sequential changes in neutrophil CD64 expression

**Fig. 1:**
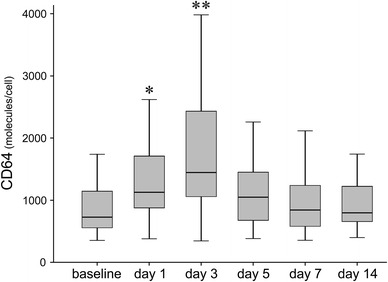
Sequential changes of neutrophil CD64 expression after surgery. Neutrophil CD64 expression elevated at day 1 after surgery, peaked at day 3, and thereafter decreased. Statistically significant differences existed between baseline level and the levels at days 1 and 3, while no significant differences existed at days 5, 7 or 14 (*<0.05, **<0.001)

The levels of neutrophil CD64 expression elevated at day 1 after surgery, peaked at day 3, and thereafter decreased: 725.0 [550.5–1,166.0] molecules/cell at baseline, 1,128.0 [861.0–1,716.5] at day 1, 1,448.0 [1,054.0–2,632.5] at day 3, 1,049.5 [671.8–1,463.5] at day 5, 844.0 [580.0–1,285.5] at day 7, and 818.0 [652.0–1,303.5] at day 14 after surgery. Statistical analysis confirmed that significant differences existed between the baseline level and the levels at days 1 and 3 (*p* = 0.01, *p* < 0.001, respectively), while no significant differences existed between the baseline levels and those at days 5, 7 or 14 (Fig. 1).

Of the total 41 cases, 31 showed their peaks at day 3 after surgery, whereas the remaining 10 cases showed different peak points: four cases at day 1, three at day 5, one at day 7 and two at day 14 after surgery. However, there were no differences among the 31 and those 10 cases in either their baseline or perioperative profiles.

In a comparison between TKA and THA, no significant differences were seen in CD64 expressions; i.e., 788.0 [583.0–1,189.0] molecules/cell and 688.5 [532.3–1,161.0] at baseline: 991.0 [716.0–1,723.0] and 1,147.5 [865.0–1,743.8] at day 1: 1,317.0 [970.0–2,111.0] and 1,947.0 [1,139.0–2,842.5] at day 3: 1,302.5 [642.5–1,576.8] and 959.0 [680.5–1,421.8] at day 5: 887.0 [587.0–1,429.0] and 820.5 [569.0–1,266.8] at day 7: 818.0 [637.0–1,427.0] and 822.5 [648.0–1,296.3] at day 14, respectively. That is, the measured levels at just day 1 and day 3 were significantly higher than baseline, no matter whether TKA or THA.

### Sequential changes in the other parameter levels

**Fig. 2:**
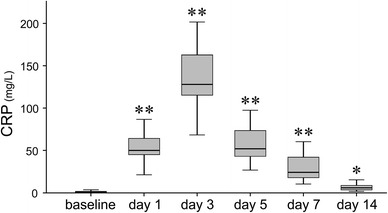
Sequential changes of C-reactive protein (CRP) after surgery CRP significantly elevated at day 1 after surgery, peaked at day 3, and thereafter tended to decrease, however, CRP still remained significantly higher compared to baseline at day 14 (*<0.05, **<0.001)

CRP significantly elevated at day 1 after surgery (*p* < 0.001), peaked at day 3 (*p* < 0.001), and thereafter tended to decrease in a manner similar to that of CD64 (Fig. 2). The level at day 14, however, still remained significantly higher compared to baseline (*p* = 0.027).

**Fig. 3:**
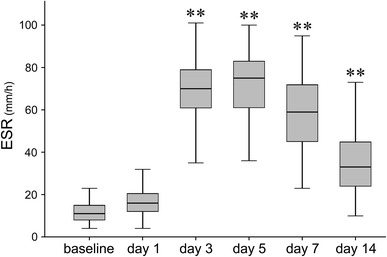
Sequential changes of erythrocyte sedimentation rate (ESR) after surgery. ESR significantly elevated at day 3 after surgery, peaked at day 5, and thereafter tended to decrease, however, like CRP, ESR still remained significantly higher compared to baseline at day 14 (**<0.001)

ESR elevated at day 3 after surgery (*p* < 0.001), peaked at day 5 (*p* < 0.001), and subsequently tended to decrease, but the level at day 14 still remained higher than baseline (*p* < 0.001) (Fig. 3).

**Fig. 4:**
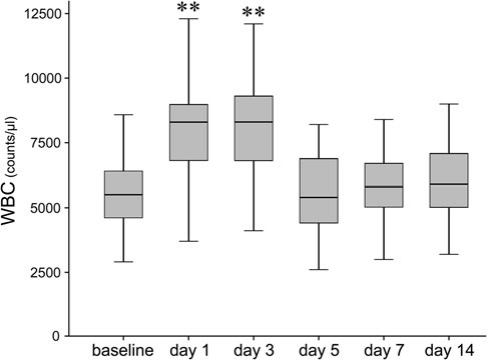
Sequential changes of white blood cell count (WBC) after surgery. WBC elevated immediately after surgery, peaked at day 3, and alleviated to near baseline at day 5 (**<0.001)

WBC elevated immediately after surgery, peaked at day 3 (*p* < 0.001 at either day 1 or 3), and alleviated at day 5 (Fig. 4).

### Analysis of the highest CD64 level

Seventeen patients showed their peak levels at over 2,000 molecules/cell, the level which had been reported to be a cutoff value for distinguishing infection [1, 2].

The highest CD64 level positively correlated with the baseline CD64 level (*r* = 0.634, *p* < 0.001). The results of the multiple regression analysis showed that the sole parameter of baseline CD64 level significantly explained the highest CD64 level (*p* < 0.001).

In a comparison between the baseline and the highest postoperative CD64 levels, a peak of 1.9 [1.6–2.7] times the preoperative level was found. This ratio was not influenced by the baseline CD64 level, the baseline demographic characteristics, or perioperative profiles.

## Discussion

In the current study, we excluded 13 patients from the analysis because of comorbidities or revision surgeries; these conditions, themselves, may influence the levels of CD64 or the other parameters.

In consequence, the results obtained from the present study demonstrated that CD64 expressions on neutrophils elevate at early post-operative timing, and that they are followed by a rapid return to nearly baseline levels by day 5 without distinction for TKA or THA. Moreover, it is worth noting that the elevation ratio of CD64 peaked at about 2 times the baseline level in all cases, even exceeding the red-flag 2,000 molecules/cell level, which has been regarded as a cutoff value for indicating local musculoskeletal infection [1, 2]. This result is reported here for the first time as far as we know.

The temporary elevation of CD64 after surgery would seem reasonable because it is consistent with the previous data which Strohmeyer [13] or Kolackova [14] reported on cardiovascular surgeries. However, the reason for this elevation is uncertain. A possibility is that it may be caused by cytokines, which are increased by surgical stress. Fjaertoft et al. [12] reported that in postoperative patients elevations of G-CSF, IL-6 and -8 levels were found from 6 to 48 h after the start of surgery, while IFN-γ was not elevated. Based on these observations, the temporary elevation of CD64 might be related to stimulation by any cytokines other than IFN-γ.

Of note is that no cases with postoperative infection complications were included in this study. This is because the primary aim of this study was not to establish definite rules for detecting infection using CD64 but to clarify the normal sequential changes of CD64. In fact, we have found no reports on CD64 expression in infected total joint arthroplasty. Accordingly, we must bear in mind that further studies, including infection-complicated cases following surgery, are needed to establish clear-cut standards for detecting postoperative infection at an early stage, or even to be able to say that if CD64 exhibited any deviant behavior from its natural course after surgery, a possibility of occurrence of infection may have to be considered.

In light of detecting infection, the other measured inflammatory markers (CRP level, ESR and WBC) have also been thought to be useful. Moreschini et al. [15] reported that CRP reached a peak on day 3 followed by a relatively rapid return to baseline levels compared with ESR, and they concluded that the sustained high level of CRP over 60 days after total joint arthroplasty suggested infection complication. Honsawek et al. [16] reported that CRP returned to within the normal range by 6 weeks after total knee arthroplasty in a case with no complications. On the other hand, Laiho et al. [17] showed that early elevation of CRP after surgery decreased to the baseline value in about 1 week. The present study, however, showed that CRP and ESR increased early, and still showed higher even at 14 days after surgery, compared with the respective baseline levels of each, while the sequential changes in WBC synchronized to those in CD64. These observations led us to consider that WBC may be as useful for detecting infection as CD64, whereas CRP and ESR may not be. However, Tanaka et al. [2] reported that the area under curve of CD64 was significantly larger than those of CRP, ESR and WBC for identification of local infection. Thus, it can be totally recognized that the sequential changes in CD64 expression are more distinguishable than those in CRP, ESR or even WBC.

The important point to note in the current study is that in all cases prophylactic antibiotics were administered during the surgeries and for 3 days following. The previous investigators, Tanaka et al. [2] pointed out that the sensitivity of CD64 for detecting infection was reduced by administration of antibiotics at the time of sampling. Thus, there is a possibility that results similar to those obtained in the current study may not be obtained when antibiotics have been administered for over 3 days after surgery.

In conclusion, the range of neutrophil CD64 level can increase to as much as 1.6–2.7 times the baseline levels, even exceeding 2,000 molecules/cell, in early post-operative days following total joint arthroplasty without cause for concern about infection, so long as it is followed by a rapid return to nearly baseline levels by day 5. The data obtained can be expected to form a possible basis for early diagnosis of postoperative periprosthetic infection.
